# Extract of *Vernonia condensata*, Inhibits Tumor Progression and Improves Survival of Tumor-allograft Bearing Mouse

**DOI:** 10.1038/srep23255

**Published:** 2016-03-24

**Authors:** Elizabeth Thomas, Vidya Gopalakrishnan, Ranganatha R. Somasagara, Bibha Choudhary, Sathees C. Raghavan

**Affiliations:** 1 Department of Biochemistry, Indian Institute of Science, Bangalore, 560 012, India; 2 Institute of Bioinformatics and Applied Biotechnology, Electronics City, Bangalore 560 100, India

## Abstract

Medicinal plants are considered as one of the ideal sources for cancer therapy due to their bioactive contents and low toxicity to humans. *Vernonia* genus is one of the common medicinal plants, which has wide spread usage in food and medicine. However, there are limited studies to explore its anticancer properties. In the current study, we have used *Vernonia condensata*, to explore its anticancer activity using various approaches. Here, we show that extract prepared from *Vernonia condensata* (VCE) exhibits cytotoxic properties against various cancer cells in a dose- and time-dependent manner. Interestingly, when treated with VCE, there was no significant cytotoxicity in peripheral blood mononuclear cells (PBMCs). Flow cytometry analysis revealed that although VCE induced cell death, arrest was not observed. VCE treatment led to disruption of mitochondrial membrane potential in a concentration dependent manner resulting in activation of apoptosis culminating in cell death. Immunoblotting studies revealed that VCE activated intrinsic pathway of apoptosis. More importantly, VCE treatment resulted in tumor regression leading to significant enhancement in life span in treated mice, without showing any detectable side effects. Therefore, for the first time our study reveals the potential of extract from *Vernonia condensata* to be used as an anticancer agent.

Cancer is considered as the most dangerous disease in humans and is a major health issue worldwide. Preclinical studies have shown that cancer development is a multifactorial and multistage process consisting of three distinct phases: initiation, promotion and progression^1^,^2^. The existing treatment modalities include radiation, chemotherapy, immunosuppression and surgery. Particularly, most of the chemicals used for therapy exhibit significant side effects. Hence, there is need for alternative agents mainly derived from natural sources to improve the chemotherapy options.

Majority of recent research focused on the use of phytochemicals including sulforaphane, resveratrol, genistein, curcumin, epigallocatechin-3-gallate (EGCG), gingerol, diallyl sulfide, brassinin and caffeic acid phenyl ester for the control and containment of tumorigenesis^3^,^4^,^5^,^6^,^7^,^8^,^9^. In addition to this, plant extracts have been used in chemoprevention and chemotherapeutic studies mainly due to the synergistic effect contributed by different plant metabolites.

*Vernonia* (Asteraceae) is the largest genus in the tribe Vernoniae, having close to 1000 species^10^ and is found generally in the tropical regions^10^. The genus *Vernonia* is known for its various uses in food, medicine and industrial applications. Moreover, the plant is rich in nutrients including amino acids, minerals and vitamins^10^,^11^,^12^,^13^,^14^. The bioactive compounds such as vernolide and water soluble peptides called edotides isolated from *Vernonia amygdalina* have shown cytotoxicity against cancer cell lines^14^,^15^,^16^,^17^,^18^. In addition, Vernonioside B2, Vernodalol, Vernodalin and Vernolide are the other major bioactive metabolites isolated from this genus, which have potent antischistosomal, antibacterial and antifungal properties^17^,^18^.

*Vernonia condensata*, the medicinal plant investigated in the present study is reportedly used for protection against snake bites^17^,^18^ and has analgesic and anti-inflammatory properties^10^. Despite the reported ethnopharmacological properties, there are no studies to test the anticancer efficacy of this species or its isolated molecules in cancer cells or in tumor animal models.

Here, we show that extract prepared from leaves of *Vernonia condensata* exhibit cytotoxic properties against various cancer cell lines by inducing apoptosis via intrinsic pathway. Besides, VCE treatment resulted in tumor regression leading to a 60% increase in the survival rate of treated mice. Thus, our study reveals for the first time, the anticancer properties of *V. condensata*.

## Results

### *Vernonia condensata* extracts induce cytotoxicity in a dose-dependent manner

**Figure 1:**
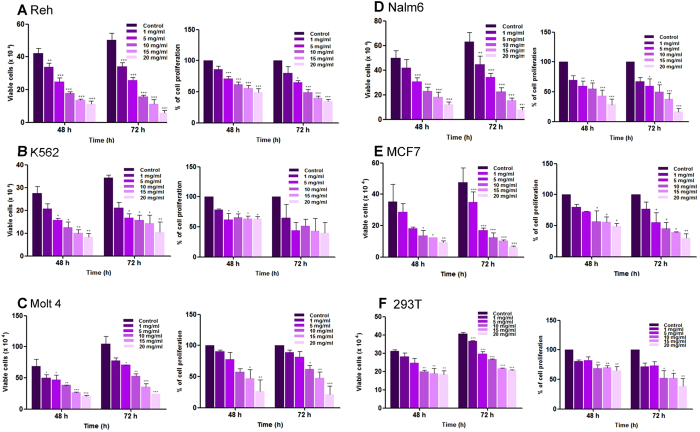
Evaluation of cytotoxic effect of *Vernonia condensata* extract (VCE) on cancer cell lines. Leukemic cell lines (Reh, K562, Nalm6, Molt4), breast adenocarcinoma cell line (MCF7) and human embryonic kidney cell line (HEK 293T) were treated with different concentrations of VCE (1, 5, 10, 15 and 20 mg/ml). In each case, trypan blue exclusion and MTT assays were performed to determine the impact of VCE treatment on cells after 48 and 72 h of treatment and data are represented as histogram. Different panels shown for cell lines are Reh (**A**), K562 (**B**), Molt4 (**C**), Nalm6 (**D**), MCF7 (**E**) and HEK293T (**F**). Data presented are based on two or more independent experiments and error bars represent S.E.M. In all panels P value represents *P < 0.05; **P < 0.01; ***P < 0.001.

In the present study, we have used a group of leukemic cell lines (Reh, Nalm6, K562 Molt4), breast cancer cell line (MCF7) and human embryonic kidney cell line (HEK293T) to evaluate the potential of extracts prepared from *Vernonia condensata*, denoted as VCE to induce cytotoxicity. A significant decrease in cell viability was observed with increasing concentration of VCE as measured by trypan blue dye exclusion assay (Fig. 1). Reh and Nalm6 cells showed highest sensitivity followed by K562 and MCF7 (Fig. 1). Among the cancer cell lines tested, Molt4 exhibited least sensitivity. Interestingly, VCE induced only limited cytotoxicity in 293T cells, compared to the cancer cell lines.

Effect of VCE on cell proliferation was further evaluated in different cancer cell lines and normal cell line using MTT assay. Results showed that the proliferation of cells was affected significantly at a concentration of 5 mg/ml. VCE exhibited maximum activity in Reh cells followed by K562, Nalm6, MCF7 and Molt4 cells after 72 h of treatment (Fig. 1). VCE induced limited effect on the proliferation of normal cell line, HEK293T. Taken together, our results showed that VCE interfered with cell proliferation in many cancer cell lines studied (Fig. 1).

Based on Trypan blue and MTT assays, Reh and Nalm6 cells exhibited highest sensitivity towards VCE with an IC_50_ of 10 and 9.0 mg/ml, respectively (Table 1). Other cancer cell lines such as K562, Molt4 and MCF7 showed an IC_50_ of 10.7, 11.4 and 11.9 mg/ml, respectively at 48 h, while it was 26 mg/ml, in case of kidney cells, 293T (Table 1).

Further, we compared the effect of paclitaxel, a plant derived anticancer compound on Reh cells. Results showed that cell viability and proliferation of the cells were significantly affected at a lower concentration of paclitaxel (Suppl. Fig. 1). Since we use extracts from *V. condensata*, direct comparison of the effective dosage between the two is not achievable.

### VCE does not induce cytotoxicity in peripheral blood mononuclear cells

Since VCE induced cytotoxicity in leukemic cells, we examined the effect of VCE on peripheral blood mononuclear cells. PBMCs were treated with increasing concentrations of VCE (1, 5, 10 and 20 mg/ml, 48 h) and cytotoxicity was determined. Results showed no or limited sensitivity on PBMCs upon treatment with VCE (IC_50_ > 20 mg/ml) suggesting that VCE did not affect normal cell viability (Suppl. Fig. 2).

### VCE induces cell death without causing cell cycle arrest

**Figure 2:**
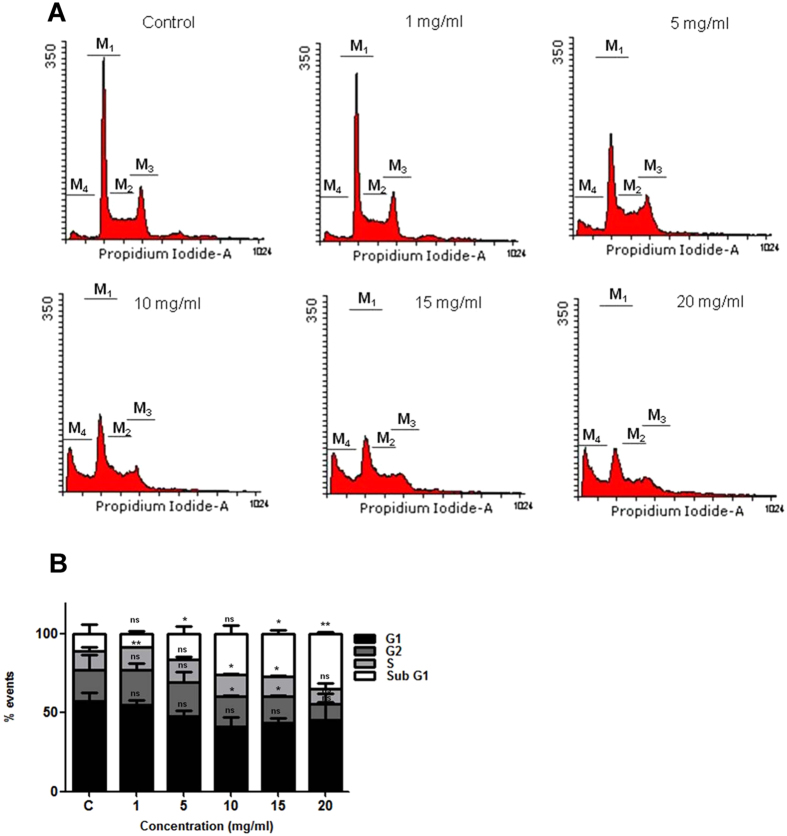
Effect of VCE on cell cycle phase distribution. (**A**) Reh cells were treated for 48 h with different concentrations of VCE (1, 5, 10, 15 and 20 mg/ml) and the cell cycle phase distribution was evaluated using flow cytometry. Each cell cycle phase is indicated as M1, M2, M3 and M4. M1 corresponds to G1 phase, M2 for S phase, M3 for G2 phase and M4 for sub G1 phase. (**B**) Bar diagram depicts the % of cells in different phases (G1, S, G2 and sub G1) of cell cycle. P value represents *P < 0.05; **P < 0.01; ***P < 0.001.

To examine whether the growth inhibition observed resulted due to cell cycle arrest or apoptosis, Reh cells were treated with increasing concentrations of VCE (1, 5, 10, 15 and 20 mg/ml) for 48 h and the cell cycle distribution was analyzed by flow cytometry following staining with propidium iodide (PI). A significant accumulation of cells in sub G1 phase was observed at a concentration of 5 mg/ml VCE onwards, which is indicative of apoptosis (Fig. 2). However, no cell cycle arrest was noted upon treatment with VCE. The result suggests that VCE induced apoptosis in Reh cells in a concentration dependent manner.

### Treatment with VCE results in decreased Mitochondrial Membrane Potential (Δψ_m_)

**Figure 3:**
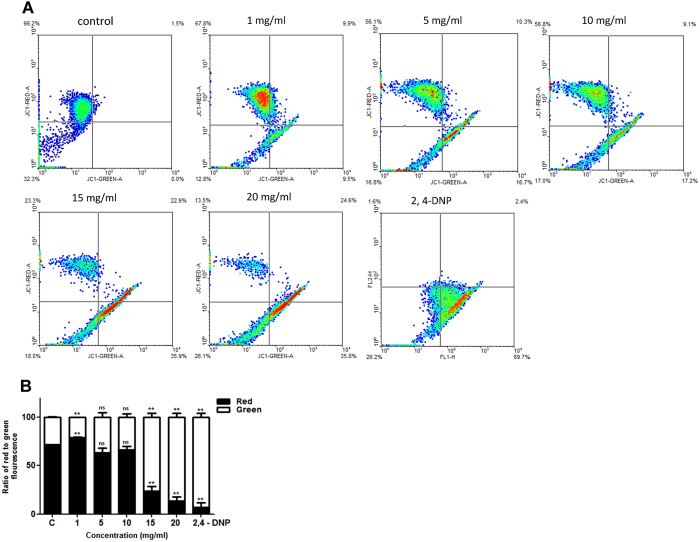
Evaluation of effect of VCE on mitochondrial membrane potential. (**A**) Reh cells were treated with increasing concentrations of VCE (1, 5, 10, 15 and 20 mg/ml) for 48 h and mitochondrial membrane potential was assayed using JC-1 staining followed by flow cytometry. Histogram shows a spectral shift from red to green upon treatment. (**B**) The ratio of red to green fluorescence is represented as bar diagram with error bars and P value represents *P < 0.05; **P < 0.01; ***P < 0.001.

Loss of mitochondrial membrane potential (MMP) is a process which precedes apoptosis^19^. We examined the change in MMP following treatment with VCE in Reh cells by flow cytometry of JC-1 stained cells (Fig. 3). The membrane potential (Δψ_m_) was measured by analysing the ratio of red to green fluorescence emitting cells following treatment with VCE. Results showed that while control cells exhibited red fluorescence indicating an unperturbed MMP, a concentration dependent increase in the green fluoresced cells was observed indicating loss of mitochondrial membrane potential upon VCE treatment (Fig. 3). Thus, our data suggests that VCE induced the disruption of MMP resulting in cytosolic accumulation of monomeric JC-1.

### VCE induces apoptosis

**Figure 4:**
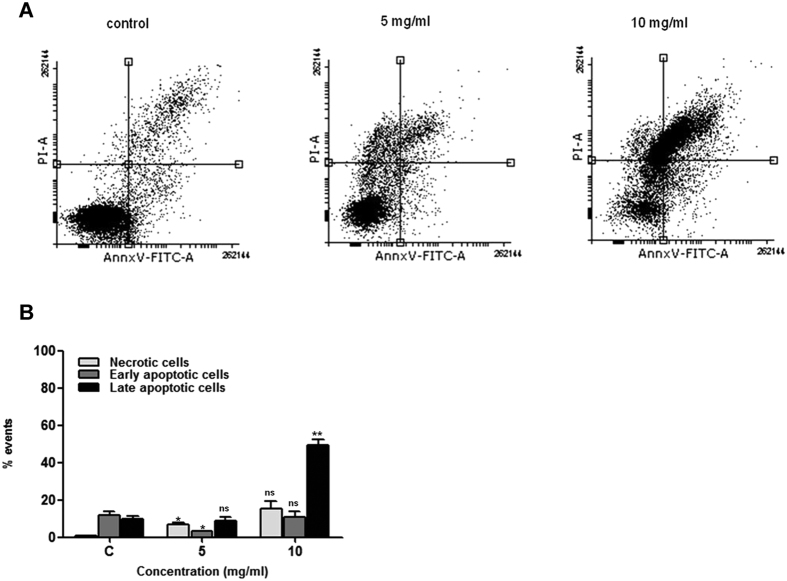
Annexin V-FITC and PI staining to evaluate apoptosis in Reh cells following VCE treatment. Reh cells were treated with VCE (5 and 10 mg/ml, for 48 h), incubated with annexin V-FITC and PI and analyzed using flow cytometry. (**A**) In each panel the lower left quadrant shows cells, which are negative for both PI and annexin V-FITC, upper left quadrant shows only PI positive cells, which are necrotic. The lower right quadrant shows annexin positive cells (early apoptotic) and the upper right quadrant shows annexin and PI positive cells (late apoptosis cells). (**B**) The percentage of necrotic, early and late apoptotic cells is represented in histogram. P value represents *P < 0.05; **P < 0.01; ***P < 0.001.

Since the above studies suggested that VCE affected the cell viability by altering mitochondrial membrane potential, we were interested in examining the induction of apoptosis by annexin V-FITC/PI staining. Reh cells were treated with VCE (5 and 10 mg/ml for 48 h) and stained using annexin V-FITC/PI. Results showed that VCE induced apoptosis in a concentration dependent manner (Fig. 4). ~8.9% cells were late apoptotic and 3.4% in early apoptotic stage upon treatment with 5 mg/ml of VCE (Fig. 4). Interestingly, 15.1, 10.8 and 49.2% were in necrotic, early and late apoptotic stages, respectively when treated with 10 mg/ml of VCE (Fig. 4). These results suggest that VCE induced translocation of phosphatidyl serine, a marker for apoptosis upon treatment. Further, the annexin V-FITC/PI stained cells indicated damage to cell membrane resulting in nuclear staining.

### VCE induces intrinsic pathway of apoptosis

**Figure 5:**
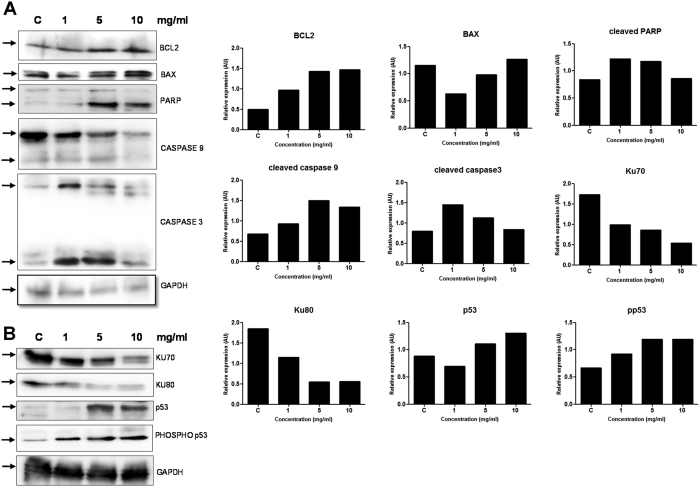
Effect of VCE on expression of different apoptotic markers. (**A,B**) Cell extract was prepared after 48 h of VCE treatment (1, 5, and 10 mg/ml) in Reh cells. Untreated cells grown for 48 h served as control. Cell extract (~30 μg) was resolved on SDS-PAGE and western blot analysis was performed using antibodies against BCL2, BAX, PARP, CASPASE3, CASPASE9, KU70, KU80, p53 and PHOSPHO-p53. GAPDH was used as the loading control.

In order to study the mechanism by which VCE induced cell death, we examined the expression levels of apoptotic markers by treating Reh cells with the increasing concentration of the extract (1, 5 and 10 mg/ml) for 48 h. Cell lysate was prepared and used for Western blotting (Fig. 5). Results showed upregulation of BCL2, an antiapoptotic protein and proapoptotic protein, BAX. PARP is a nuclear polymerase involved in DNA repair in response to environmental stress. Cleavage of PARP by CASPASE3 is important in facilitating the cellular disassembly and serves as a marker for cells undergoing apoptosis. Interestingly, we observed PARP cleavage at higher concentrations of VCE treatment (Fig. 5A). We also observed an increase in the cleavage of CASPASE9, an indicator of intrinsic pathway of apoptosis, particularly at a concentration of 5 mg/ml (Fig. 5A), although such an increase was not evident at the highest concentration as we could recover only less number of cells for analysis due to increased cell death. Another marker of apoptosis, CASPASE3 also showed an increased cleavage at 1 and 5 mg/ml of VCE, further indicating activation of apoptosis (Fig. 5A). Since the observed apoptosis was dependent on CASPASE3, expression of p53 and PHOSPHO-p53 (p-p53), a known activator of apoptosis was investigated. Results showed a remarkable, concentration dependent increase in p53 expression and its activation to p-p53 (Fig. 5B). A simultaneous decrease in the levels of KU70 and KU80 proteins, which are involved in double-strand break repair was also evident which could result in accumulation of DNA damage and hence apoptosis (Fig. 5B). Taken together our results suggest that apoptosis induced during VCE treatment is dependent on the intrinsic pathway.

### Administration of VCE in mice leads to inhibition of tumor progression

Ehrlich ascites carcinoma cells (EAC) were used for induction of tumor in Swiss albino mice for investigating the anticancer potential of VCE. Based on preliminary study a concentration of 50 mg/kg b.wt was used for evaluating the anti-tumour property of VCE (data not shown).

**Figure 6:**
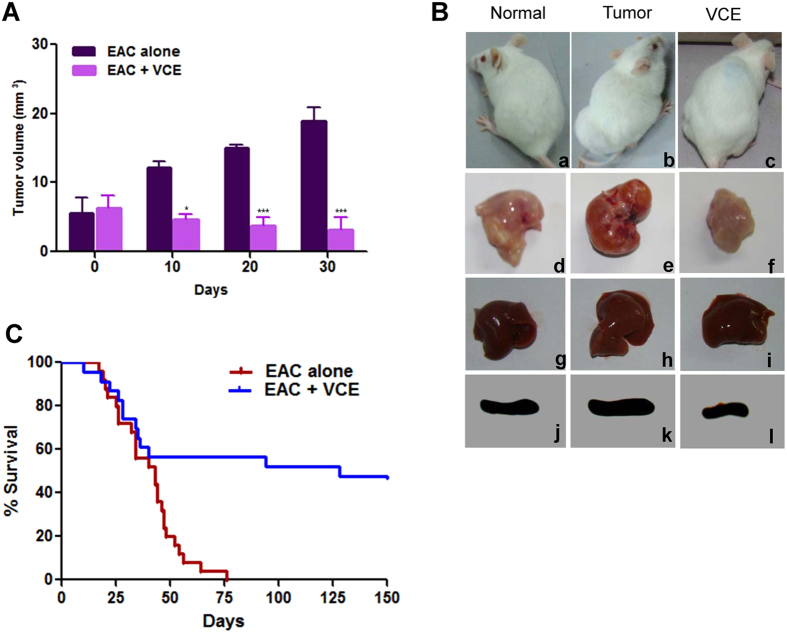
Effect of VCE on progression of solid tumor in mice. Solid tumor was induced in Swiss albino mice by injecting EAC cells. Six doses of VCE (50 mg/kg.b.wt.) were administered to tumor bearing mice every alternate day from 12^th^ day of EAC cell injection. (**A**) Tumor volume was measured following VCE treatment at different time points upto 30^th^ day and shown along with untreated control. A total of 24 animals comprising 8 animals in each group were used in triplicates for the study. Error bars indicate standard error of mean based on three independent experiments. P value represents *P < 0.05; **P < 0.01; ***P < 0.001. (**B**) The gross appearance of different organs of normal animals without tumor, control animals bearing tumor and VCE treated tumor animals on 25^th^ day. a) mouse with no tumor, b) mouse bearing tumor, c) tumor bearing mouse after treatment with VCE, d) thigh tissue of normal mouse, e) tumor tissue, f) thigh tissue of the VCE treated mouse, g) liver from normal mouse, h) liver of a tumor mouse, i) liver from VCE treated mouse, j) spleen of a normal mouse, k) spleen of a tumor bearing mouse, l) spleen of VCE treated mouse. (**C**) Kaplan–Meier survival curves of mice treated with VCE and untreated control mice bearing tumor. 8 animals per group in triplicates were used for the study.

On 12^th^ day of EAC injection, the mice bearing tumor were treated with six doses of VCE (50 mg/kg b.wt) with an interval of two days in between the doses. Interestingly, there was a significant decrease in tumor growth upon treatment with VCE unlike in untreated tumor bearing mice (Fig. 6A,B). An average tumor volume of 12.1, 15.0 and 18.9 mm^3^ on 10^th^, 20^th^ and 30^th^ day, respectively was observed in case of untreated mice, while upon VCE administration, the average tumor size was reduced to 4.65, 3.74 and 3.12 mm^3^, respectively (Fig. 6A). Furthermore, the gross appearance of thigh tissues of tumor bearing mice treated with VCE versus those taken from untreated mice showed significant tumor regression when analyzed on 25^th^ day (Fig. 6Ba–f). While liver and spleen from tumor control mice showed alterations in morphology compared to that of normal mice, the normal morphology was significantly restored in VCE treated animals. Thigh tissue, liver and spleen of a normal animal served as control (Fig. 6B). Thus, our study suggests that VCE has the potential for reducing tumor growth in mice models significantly.

### VCE enhances the survival of tumor bearing mice and reduces tumor burden by inducing apoptosis

We observed that survival time of VCE treated tumor bearing animals was significantly increased as compared to that of untreated tumor animals (Fig. 6C). While 83% (20/24) of the tumor bearing mice (tumor control) were dead by 50^th^ day of tumor development, only 42% (10/24) died after VCE treatment (Fig. 6C). Importantly, 50% of VCE treated mice survived more than 130 days in contrast to untreated tumor controls, which survived only upto 75 days (Fig. 6C). Hence, our results suggested that VCE treatment caused tumor regression and significantly increased the survival rate in mouse tumor models.

**Figure 7:**
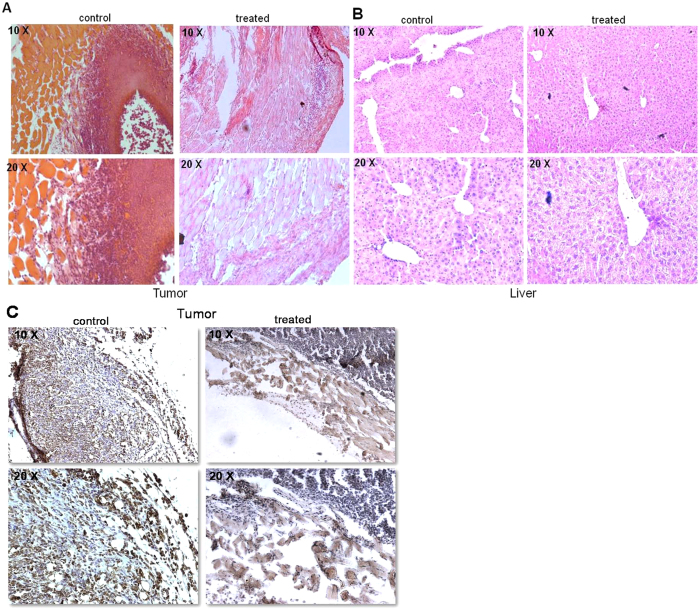
Histological and immunohistochemical analysis of VCE treated tumor mice. (**A,B**) Haematoxylin and Eosin (HE) stained sections prepared from either tumor tissues (**A**) or liver (**B**), which were from VCE treated mice for 25 days or untreated tumor control animals. Images represent HE stained sections of control tumor and VCE treated tumor (**A**); control liver and VCE treated liver (**B**). (**C**) Antibody staining for Ki67 on 25^th^ day tumor tissue and VCE treated tumor tissue. Images are shown in both 10× and 20× magnification.

Further, we were interested in understanding the mechanism of tumor regression in mice tumor models using both histology and immunohistochemistry (IHC). Haematoxylin-eosin (HE) staining showed a significant reduction in infiltrated tumor cells in the thigh tissues of tumor bearing mice following VCE treatment, as compared to untreated controls (Fig. 7A). Unlike liver sections from tumor control mice, those from treated mice revealed presence of large round nuclei, which were comparable to that of normal mice (Fig. 7B). In addition, IHC experiments revealed the presence of Ki67 positive cells, which are indicative of cell proliferation in untreated tumor tissues. However upon VCE treatment, a significant decrease in tumor cell population stained with Ki67 was observed (Fig. 7C).

Taken together, we conclude that VCE induced cytotoxicity by inducing apoptosis via intrinsic pathway in various cancer cells and inhibited tumor progression leading to significant increase in the survival rate in mice. Thus, our results suggest that VCE could be developed as a potent chemotherapeutic agent.

## Discussion

It is believed that traditional herbal medicine plays a critical role in the treatment of life threatening and chronic diseases. Among those, cancer is one of the diseases where several plant products are exploited for developing effective therapeutic drugs. For this purpose, diverse medicinal plants are used in developing potent therapeutic and management interventions.

Systematic analysis of natural products as sources of anticancer agents has been done for several years. However, there is only limited success so far due to various reasons, although it is widely believed that the use of natural products may develop into one of the most successful strategies to treat cancer.

In the present study, one of the less explored plant species, *Vernonia* was investigated for its anticancer properties. The routine consumption of *Vernonia* species as a green leafy vegetable in West and Central Africa suggests the importance of this species as a source of nutrition and medicine^10^. However, despite possessing medicinal properties, there are only two studies to explore potential cytotoxic effects in cancer cells, using *Vernonia amygdalina* and *Vernonia cinerea*^20^,^21^.

In the current investigation, we show that extracts prepared from *Vernonia condensata* possess activity that can lead to cytotoxicity in various cancer cell lines tested in a concentration dependent manner, although with varying efficiency. Moreover, VCE exhibited no cytotoxic effects on peripheral blood mononuclear cells, reinforcing its therapeutic potential. Although there was no cell cycle arrest observed upon VCE treatment, it caused significant accumulation of cells in sub G1 phase (Fig. 2). In addition to this, translocation of membrane phosphatidylserine (PS) from the inner side of the plasma membrane to the surface, indicating activation of apoptosis^22^, was observed upon treatment with VCE. Although majority of the cells were dead due to apoptosis, a minor population of the cells underwent necrosis (Fig. 4). Besides, the observed concentration dependent increase in the green fluoresced cells following JC-1 staining indicated the loss of mitochondrial membrane potential following VCE treatment (Fig. 3). Hence, the extracts of *Vernonia condensata* induced cancer cell death by activating apoptosis and to a limited extent, necrosis.

The loss of mitochondrial membrane potential and Cytochrome C release followed by Caspase cascade activation are the crucial parameters for defining the role of mitochondria during apoptosis^23^. Since the alteration in MMP was observed, expression of BCL2 family proteins such as antiapoptotic BCL2 and the proapoptotic protein, BAX are expected to be altered^24^. Interestingly, a distinct increase in the level of BAX was evident following VCE treatment, which is consistent with activation of apoptosis. However, it is unusual to have upregulation of antiapoptotic protein, such as BCL2 following treatment with VCE, which needs to be investigated further. Caspase family plays a major role in apoptosis through the proteolysis of specific targets^25^. Mitochondrial pathway of apoptosis results in the activation of CASPASE3 subsequently resulting in the cleavage of PARP^23^. Interestingly, VCE treatment resulted in the activation of CASPASE9 and CASPASE3, which subsequently induced PARP cleavage (Fig. 5), suggesting the induction of apoptosis via intrinsic pathway.

Terpenoids account for most of the biological activity described so far in *Vernonia* genus, which are comprised of approximately 103 bioactive compounds^10^. Among these Vernolepin and Vernodalin purified from *Vernonia amygdalina* possess antiplatelet and cytotoxic activity. Vernolide A and B from *Vernonia cinerea* have shown potential against cancer and inflammatory conditions. Studies have suggested Vernolide A as the most promising bioactive compound identified from *Vernonia sp* that has the potential to be developed as an anticancer agent^10^. However, there are no reports so far on isolation of bioactive compounds from *Vernonia condensata*.

EAC cells are used commonly for inducing tumors in mice, and for estimating anti-cancer activity of small molecules *in vivo*^25^,^26^. They have specific characteristics such as high transplantable capability, rapid proliferation and 100% malignancy^27^. Using allograft mice model system, we demonstrated that VCE could inhibit the tumor progression in this breast adenocarcinoma model and caused significant increase in the survival of treated mice. These results in conjunction with the *ex vivo* studies suggested that the VCE contains bioactive compounds, which are capable of affecting the proliferation of cancer cells. Further, the histological examination of the tumor and liver tissue showed that treatment with VCE resulted in the recovery of morphological features that were disrupted due to tumor growth. Immunohistochemical studies for Ki67 also confirmed a reduction in cell proliferation in VCE treated tissue, further confirming regression of tumor in mice models.

## Conclusion

Our study demonstrated that *Vernonia condensata* induces cytotoxicity in different cancer cells mainly by activating apoptosis via intrinsic pathway. Besides, VCE inhibited the tumor progression in breast adenocarcinoma mice model and significantly increased the survival rate of tumor bearing mice. Thus, *Vernonia condensata* holds promise for the development of a therapeutic agent against cancer. However, more studies are required to test for anticancer activity of VCE in advanced tumor models as well as to identify the factors that contribute towards its activity.

## Methods

### Cell lines and culture

Human pre B-cell leukemic cell lines, Reh and Nalm6, human chronic myelogenous leukemic cell line, K562, T-cell leukemic cell line, Molt4 and human breast adenocarcinoma cell line, MCF7, human embryonic kidney cell line, HEK293T and peripheral blood mononuclear cells (PBMCs) were cultured in RPMI 1640 or MEM or DMEM (HiMedia, India) supplemented with 10% heat-inactivated fetal bovine serum (Gibco BRL, USA), 100 U of Penicillin-Streptomycin/ml. Cell were cultured at 37 °C in presence of 5% CO_2_ in a humidified incubator.

### Chemicals and reagents

Chemicals and reagents were purchased from Sigma Aldrich, USA and SRL, India. Antibodies were procured from Santa Cruz Biotechnology, USA and Cell Signalling Technology, USA.

### Preparation of *Vernonia condensata* extracts (VCE)

*Vernonia condensata* plant was procured from herbal home garden, Kottayam, Kerala, India. Leaves were dried in shade, powdered and extract was prepared by resuspending the powder (300 mg) in water and by gentle mixing in shaker for 24 h followed by centrifugation (3000 rpm for 10 min). The supernatant was used for studies, which is abbreviated as VCE. Although the powder was weighed and used for extract preparation, the actual concentration of VCE would be less than what is indicated as only water-soluble metabolites were taken into account.

### Trypan blue dye exclusion assay

The effect of VCE on viability of leukemia (Reh, Nalm6, K562 and Molt4), breast adenocarcinoma (MCF7) and human embryonic kidney cell line (HEK293T) was determined by trypan blue dye exclusion assay as described^28^. Briefly, cells were seeded (0.75 × 10^5^ cells/ml) and incubated with VCE (1, 5, 10, 15 and 20 mg/ml). After 48 and 72 h of treatment, number of viable cells was determined using trypan blue staining. In case of PBMCs, cytotoxicity was measured 48 h after VCE (1, 5, 10 and 20 mg/ml) treatment. Each experiment was performed atleast twice and standard error of mean was determined and presented.

### MTT assay

The MTT assay was performed as described previously^29^. Reh, Nalm6, K562, Molt4, MCF7 and HEK293T cells were treated with VCE (1, 5, 10, 15 and 20 mg/ml), for 48 and 72 h and cell proliferation was evaluated using MTT assay. Experiment was repeated a minimum of two times with independent duplicate reactions and data are depicted as bar diagram with error bars.

### Analysis of cell cycle progression by flow cytometry

Cell cycle analysis was performed as described previously^30^. Reh cells were treated with VCE (1, 5, 10, 15, 20 mg/ml for 48 h). The cells were harvested, fixed, treated with RNase A (50 μg/ml) and stained with propidium iodide (10 μg/ml). Cellular DNA content was analyzed using flow cytometry (FACS Canto II, BD Biosciences, USA). ~10,000 cells were acquired for each analysis, and results were analysed using Flowing software (version2) and displayed as histogram.

### Determination of mitochondrial membrane potential

Cells stained with JC-1 dye were used for determining changes in mitochondrial transmembrane potential^19^,^31^. Briefly, Reh cells were treated with VCE (1, 5, 10 and 20 mg/ml), harvested (48 h) and incubated with JC-1 dye (5,5′,6,6 tetra chloro-1,1′,3,3′-tetraethylbenzimidazol-carbocyanine iodide). The stained cells were then analyzed on a flow cytometer using Cell Quest pro software with an excitation at 488 nm laser and emission at 530 nm. JC-1 monomers emit at 530 nm (red) and J-aggregates emit at 590 nm (green). The ratio of cells emitting red to green fluorescence was evaluated for each dose and plotted. 2, 4-Dinitrophenol-treated cells (2, 4-DNP) served as positive control.

### Detection of apoptosis by Annexin V-FITC/PI double-staining

One of the earlier events of apoptosis includes translocation of membrane phosphatidylserine (PS) from the inner side of the plasma membrane to the cell surface. Annexin V-FITC/PI staining was used for the quantitation of early and late apoptotic cells as described earlier^32^. Reh cells were treated with VCE (5 and 10 mg/ml for 48 h), stained with annexin V- FITC (0.2 mg/ml) and PI (0.05 mg/ml) for 20 min and were examined by flow cytometry (FACS Calibur, BD Biosciences) using Cell Quest pro software at an excitation with 488 nm laser and emission at 530 nm. A minimum of 10,000 cells was analysed per sample and illustrated as dot plot using Flowing software (version2).

### Immunoblotting

Cell extract was prepared from Reh cells after VCE treatment (1, 5, 10, mg/ml for 48 h) and western blotting was performed as described^32^. Briefly, ~30 μg of protein sample was electrophoresed on SDS-PAGE (8–10%), transferred to PVDF membrane (Millipore, USA) and probed with appropriate primary and biotinylated secondary antibodies. The primary antibodies used were BCL2, BAX, PARP, CASPASE3, CASPASE9, KU70, KU80, p53, p-p53 and GAPDH (loading control). The blots were developed using chemiluminescent reagents (Immobilon TM western, Millipore, India) and scanned by gel documentation system (LAS 3000, Fuji, Japan).

### Experimental animals

Swiss albino mice of body weight 18–24 g (8–10 weeks old) were purchased from central animal facility, Indian Institute of Science, India and maintained in polypropylene cages for the study. The animals were fed with standard pellet diet (Agro Corporation Pvt. Ltd., Bangalore, India) and water *ad libitum* and maintained under controlled conditions of temperature and humidity with a 12 h light/dark cycle. A pellet diet contains 21% protein, 5% lipids, 4% crude fiber, 8% ash, 1% calcium, 0.6% phosphorus, 3.4% glucose, 2% vitamin, and 55% nitrogen-free extract (carbohydrates). In accordance with Indian National Law on animal care and use, the animals were maintained abiding all principles and guidelines of the ethical committee for animal care, Indian Institute of science. The Institutional Animal Ethics Committee (ref. CAF/Ethics/248/2011) of Indian Institute of Science, Bangalore, India approved the experimental design of the present study.

### Preparation of Ehrlich Ascites Carcinoma (EAC) cells and induction of tumor in mice

EAC cells were withdrawn from the donor mice and resuspended in sterile phosphate buffered saline (PBS). Viable cells (1 × 10^6^ cells/22 g b. wt) were injected into the peritoneal cavity of each recipient mouse and allowed to multiply. The tumor cells were withdrawn after 8–10 days of inoculation, diluted in saline, counted and injected (1 × 10^6^ cells/animal) in left thigh of animals for developing solid tumor.

### Evaluation of the anticancer property of VCE in mouse models

Three groups of mice per batch (total 3 batches) comprising 8 animals each were used in the present study. Solid tumor was developed by injecting EAC cells as specified in 16 animals. Group I animals which received no treatment served as untreated control (normal). Group II animals that received EAC cells without any treatment served as tumor control. After 12 days of tumor cells injection, the group III animals bearing EAC solid tumors received six doses of VCE (50 mg/kg b.wt; using gastric gavage) orally with an interval of two days that continued throughout the experimental period.

Tumor size for group II and III animals were measured using vernier calipers on every alternate day and volume of tumor was determined using the formula, V = 0.5 × a × b^2^, where ‘a’ and ‘b’ are the major and minor diameters^26^,^32^,^33^. On the 25^th^ day of experimental phase, one animal from each group (normal, tumor control and VCE treated) was sacrificed and liver, spleen and thigh tissues were dissected out and processed for histological examination^26^,^32^,^33^.

### Evaluation of the survival time of tumor bearing mice after treatment with VCE

The percentage of increased survival time of treated tumor mice was measured and compared with the untreated mice bearing EAC tumor. 8 animals per group in triplicates were used in the study. The experimental animals (VCE treated) were monitored till 130^th^ day. The survival of control and experimental mice was calculated using the formula (T − C)/C × 100, where ‘T’ indicates the number of days where the treated tumor bearing animals survived and ‘C’ indicates the number of days the untreated tumor bearing animals survived^26^,^32^,^33^.

### Histological evaluation

Tumor and liver tissues of untreated and VCE treated mice were dissected out and processed as per standard protocols^26^,^32^,^33^. Briefly, the tissues were embedded in paraffin wax, sectioned at 5–10 μm in a rotary microtome (Leica Biosystems, Germany) and stained using haematoxylin and eosin. Each section was observed under light microscope and images were captured (Zeiss, Germany).

### IHC (Immunohistochemical) analysis

IHC analysis of tumor tissues was carried out using sections from control and VCE treated tumor tissues as described earlier^34^. Antibody staining was performed on formalin fixed, paraffin embedded tissues, which were sectioned with 5 μm thickness. Deparaffinized slides were rehydrated and treated with 3% H_2_O_2_ in PBS. Antigen retrieval was done using 0.01% sodium-citrate buffer followed by blocking in PBST containing 0.1% BSA and 10% FBS. Following incubation in primary antibody (Ki67-1:100; overnight at 4 °C), slides were washed and incubated with biotinylated secondary antibody (1:200, 1 h). Sections were then washed, incubated in streptavidin-HRP (1:500) and finally, sections were developed using DAB, H_2_O_2_, counterstained with haematoxylin, mounted in DPX and images were captured using a light microscope (Zeiss, Germany).

### Statistical analysis

The statistical analyses were performed using one-way ANOVA followed by Student ‘t’ test utilizing Graph Pad software prism 5.1 and values are expressed as mean ± SEM for control and experimental samples. The values were considered statistically significant, if the P value was less than 0.05.

## Additional Information

**How to cite this article**: Thomas, E. *et al*. Extract of *Vernonia condensata*, Inhibits Tumor Progression and Improves Survival of Tumor-allograft Bearing Mouse. *Sci. Rep*. **6**, 23255; doi: 10.1038/srep23255 (2016).

## Supplementary Material

Supplementary Information

## Figures and Tables

**Table 1:** IC_50_ value of VCE in various cancer cell lines and HEK293T cells.

Cell lines	IC_50_ (mg/ml) 48 h		Average of IC^50^ (mg/ml)
	Trypan blue	MTT	
Reh	7.5	13.0	10.0
K562	8.2	13.2	10.7
Molt4	10.6	12.3	11.4
Nalm6	8.8	9.2	9.0
MCF7	7.9	15.8	11.9
HEK293T	22.0	29.7	26.0
