# Supplementary material for: Extract of Vernonia condensata, Inhibits Tumor Progression and Improves Survival of Tumor-allograft Bearing Mouse
Source: Sci Rep. 2016 Mar 24;6:23255. doi: 10.1038/srep23255 (PMC4806354; doi:10.1038/srep23255)
Supplement: Supplementary Information [file srep23255-s1.pdf]

Extract of *Vernonia condensata*, Inhibits Tumor Progression and Improves Survival of Tumor-allograft Bearing Mouse

Elizabeth Thomas[1], Vidya Gopalakrishnan[2], Ranganatha R. Somasagara[1], Bibha Choudhary[2] and Sathees C. Raghavan[1]*

[1]Department of Biochemistry, Indian Institute of Science, Bangalore, 560 012, India,

[2]Institute of Bioinformatics and Applied Biotechnology, Electronics City, Bangalore 560 100, India

*Corresponding author : Ph. 091 80 2293 2674; Fax: 091 80 2360 0814

e-mail : sathees@biochem.iisc.ernet.in

Supplementary figure legends

Suppl. Figure 1. Evaluation of cytotoxic effect of Paclitaxel on Reh cell line. Reh cells were treated with different concentrations of Paclitaxel (1, 5, 10, 50, 100 nM). Cytotoxicity was monitored after 48 h of treatment by trypan blue dye exclusion and MTT assays. Data presented are based on two independent experiments and error bars are indicated. P value indicates *P<0.05; **P<0.01; ***P<0.001.

Suppl. Figure 2. Evaluation of cytotoxic effect of *Vernonia condensata* extract (VCE) on peripheral blood mononuclear cells (PBMC). PBMCs were treated with different concentrations of VCE (1, 5, 10 and 20 mg/ml). Trypan blue exclusion assay was performed after 48 h of treatment and data is represented as histogram.

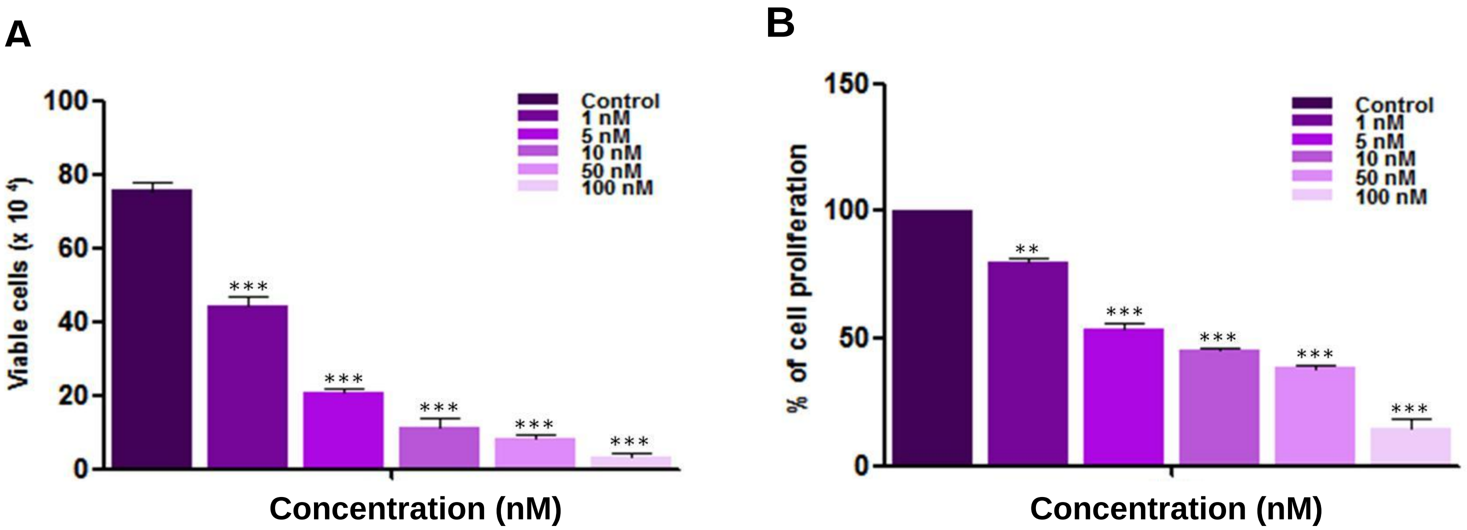

Supplementary Figure 1

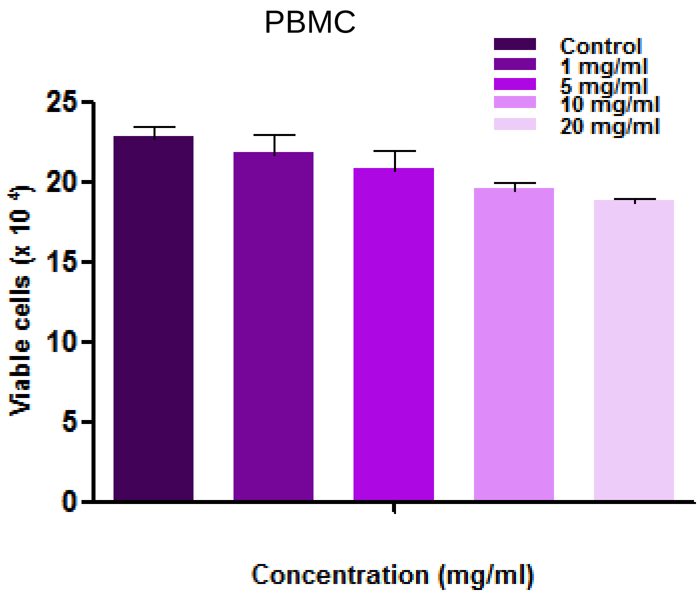

Supplementary Figure 2
